# Automatic detection and quantification of antimicrobial inhibition zones using YOLO11n with *post-hoc* interpretability validation

**DOI:** 10.3389/fmicb.2026.1810754

**Published:** 2026-04-28

**Authors:** Fatih Ciftci, Azime Erarslan, Javad Rahebi

**Affiliations:** 1 Department of Biomedical Engineering, Faculty of Engineering, Fatih Sultan Mehmet Vakif University, Istanbul, Türkiye; 2 Biomedical Electronic Design Application and Research Center (BETAM), Fatih Sultan Mehmet Vakif University, Istanbul, Türkiye; 3 BioriginAI Research Group, Department of Biomedical Engineering, Fatih Sultan Mehmet Vakif University, Istanbul, Türkiye; 4 Department of Bioengineering, Faculty of Chemical and Metallurgical Engineering, Yildiz Technical University, Istanbul, Türkiye; 5 Department of Electrical and Electronics Engineering, Istanbul Topkapi University, Istanbul, Türkiye

**Keywords:** antimicrobial susceptibility testing, categorical agreement, clinical microbiology, deep learning, Grad-CAM, inhibition zone measurement, YOLO11n

## Abstract

**Introduction:**

The escalating prevalence of antimicrobial resistance (AMR) constitutes a global healthcare crisis, necessitating rapid and standardized diagnostic solutions for antimicrobial susceptibility testing (AST). This study introduces an advanced, end-to-end artificial intelligence framework designed for the fully automated detection, quantification, and clinical interpretation of inhibition zones from disk diffusion assays using the state-of-the-art You Only Look Once (YOLO11n) object detection model.

**Methods:**

A high-resolution dataset of Petri dish images, featuring antibiotic discs tested against *Escherichia coli, Salmonella*, and *Staphylococcus* aureus, was curated to train and validate the system under standardized conditions. The proposed pipeline localizes inhibition zones, quantifies their diameters with sub-millimeter precision, and automates resistance classification by integrating dynamic Clinical and Laboratory Standards Institute (CLSI) breakpoint criteria. Model interpretability was further ensured through Grad-CAM visualizations.

**Results:**

Evaluation results demonstrate that the YOLO11n-based system achieved a Categorical Agreement (CA) of 94.2%, with a Very Major Error (VME) rate of 1.2% and a Major Error (ME) rate of 1.8%, performing well within clinical safety thresholds. High spatial accuracy was confirmed by a correlation coefficient of *R*^2^ = 0.98 and a Mean Absolute Error (MAE) of 0.42 mm in zone diameter prediction.

**Discussion:**

Grad-CAM analysis confirmed that the architecture's attention is consistently aligned with biologically relevant inhibition boundaries rather than background artifacts. By providing an objective, reproducible, and transparent method for digital antibiogram analysis, this framework offers significant potential for seamless integration into clinical microbiology workflows and large-scale AMR monitoring programs.

## Graphical Abstract

**Figure:**
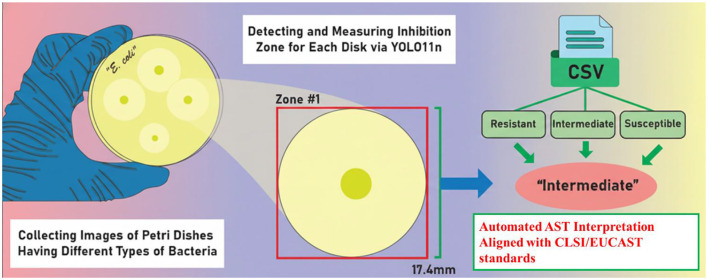

## Highlights

- An end-to-end AI-based pipeline was developed for the fully automated detection and interpretation of disk diffusion antimicrobial susceptibility tests (AST).
- The state-of-the-art YOLO11n architecture ensures rapid and accurate localization of inhibition zones with sub-millimeter spatial precision.
- The system achieved a Categorical Agreement (CA) of 94.2% and a Very Major Error (VME) rate of 1.2%, significantly outperforming clinical safety thresholds.
- Inhibition zone diameters are automatically quantified and classified (S/I/R) by integrating dynamic CLSI 2024 (Gaur et al., 2023; Hombach et al., 2013) breakpoint criteria via an automated lookup framework.
- Model interpretability is validated through Grad-CAM analysis, ensuring diagnostic transparency and focus on biologically relevant inhibition boundaries.

## 1 Introduction

Traditional antimicrobial susceptibility testing (AST) techniques, such as manual disk diffusion analysis, are often time-consuming, subjective, and prone to human error (Salam et al., 2023). With the integration of artificial intelligence, these processes can be automated to deliver faster, more reliable, and highly reproducible results (Haibe-Kains et al., 2020; Naugler and Church, 2019). In recent years, artificial intelligence (AI) has become an indispensable asset in microbiological diagnostics, offering rapid, reproducible, and objective alternatives to traditional manual image analysis (Alsulimani et al., 2024; Chaturvedi et al., 2024). AST, particularly the disk diffusion method, remains one of the most widely used techniques for classifying bacterial resistance (Gajic et al., 2022). However, conventional interpretation relies heavily on manual measurement of inhibition zones, which introduces variability, consumes valuable time, and is susceptible to human error (Lestari et al., 2008).

The integration of AI, specifically through machine learning (ML) and deep learning (DL) models, enables high-throughput and standardized analysis of inhibition zones in Petri dish images (Orrell-Trigg et al., 2024). These models are capable of learning from annotated datasets to identify and quantify relevant features with high consistency and precision (Gong et al., 2023). Deep learning approaches, leveraging layered neural network structures, making them well suited for tasks such as inhibition zone detection, antibiotic disc localization, and susceptibility classification (Popa et al., 2022).

In the context of AST automation, deep learning frameworks can process Petri dish images to identify antimicrobial disks, detect the corresponding inhibition zones, and measure their diameters with minimal human intervention (Alonso et al., 2017). This significantly enhances the speed and reliability of diagnostic workflows. By comparing measured zone diameters against established interpretive criteria, these systems can accurately classify bacterial strains as resistant, intermediate, or susceptible (Hombach et al., 2013; Gaur et al., 2023). Moreover, unlike traditional manual assessments, AI-driven models enable consistent interpretation across laboratories, reducing operator-dependent variability.

Among the various deep learning architectures, Convolutional Neural Networks (CNNs) have demonstrated strong performance in biomedical image analysis by capturing hierarchical spatial patterns (Popa et al., 2022; Khan et al., 2020). Nevertheless, standard CNNs often encounter limitations in tasks requiring simultaneous localization and classification, especially when dealing with crowded or low-contrast regions. The You Only Look Once (YOLO) architecture addresses these limitations by reformulating object detection as a single-stage regression problem (Gallagher and Oughton, 2025). It predicts both class labels and spatial coordinates in a single pass, enabling real-time, high-accuracy detection of antimicrobial disks and their surrounding inhibition zones (Mairi et al., 2025).

Recent research has increasingly focused on automating AST to enhance measurement accuracy and diagnostic efficiency. A recent study introduced a YOLO-based deep learning framework capable of detecting Petri dishes, antibiotic disks, and inhibition zones, achieving high precision in diameter estimation and further integrating results into a mobile application for real-time analysis (Gullu et al., 2024). Another investigation emphasized the importance of automation in minimizing inter-technician variability, highlighting how computer vision methods can enable standardized, reproducible, and high-throughput measurement of inhibition zones in disk-diffusion assays (Balmages et al., 2025). A separate approach leveraged a laser speckle imaging technique, combined with subpixel correlation and machine learning, to distinguish microbial activity in early time windows, providing near-real-time assessment of antimicrobial effects (Li et al., 2024). Additionally, traditional image processing techniques using color thresholds and size references were combined with classification models to interpret antibiograms, demonstrating the practical feasibility of automated zone analysis (Suponenkovs et al., 2024). Collectively, these studies underscore the growing integration of AI and image analysis in AST, aiming to streamline interpretation and improve reliability in clinical microbiology.

While recent platforms like the AI-based mobile application by (Pascucci et al. 2021) and the web-based “Disc Diffusion Reader” by (Nguyen et al. 2025) have advanced digital AST, they often function as “black-box” systems or require cloud dependency. Our work addresses these gaps by implementing a locally deployable YOLO11n-based pipeline that integrates explainable AI (Grad-CAM) and automated interpretive logic aligned with current CLSI/EUCAST standards. The implementation of Explainable AI (XAI) via Grad-CAM provides a transparent validation framework, bridging the gap between deep learning outputs and clinical expert intuition by confirming the model's spatial focus.

This study introduces a fully automated, AI-driven framework for the detection and classification of antimicrobial inhibition zones using the YOLOv11n object detection model. Unlike traditional image processing or manual measurement techniques, the proposed method offers a scalable and objective solution for analyzing disk diffusion assay plates by directly extracting inhibition zone dimensions from standardized photographs. Through high-quality image acquisition, careful annotation, and rigorous model training, the system achieves precise measurement of inhibition diameters and maps these values to established CLSI breakpoints to determine susceptibility. By incorporating Grad-CAM visualizations for model interpretability and validating performance across three clinically relevant bacterial species *E. coli, Salmonella*, and *S. aureus* this work demonstrates the potential of real-time, deep learning-based AST tools to improve diagnostic consistency and reduce manual workload in microbiology laboratories.

## 2 Methods

We developed an AI-based system to automatically detect and quantify antimicrobial inhibition zones on disk diffusion assay plates using the YOLOv11n object detection architecture. The goal was to generate an objective, reproducible, and rapid measurement of inhibition areas directly from high-resolution photographs of petri dishes, eliminating the need for manual measurement. The workflow included systematic dataset acquisition, image standardization to ensure real-world applicability, manual annotation of inhibition zones as a single object class, model training and optimization, and post-inference visualization with Grad-CAM to validate biological relevance. In this study, Grad-CAM was utilized as a *post-hoc* diagnostic tool to visually audit the YOLO11n model. While Grad-CAM does not influence the millimetric calculation or the S/I/R classification process, it serves as a critical quality control layer to ensure that the model's feature extraction is grounded in the biological characteristics of the inhibition zones. Finally, inhibition zone diameters predicted by YOLOv11n were compared against Clinical and Laboratory Standards Institute (CLSI) interpretive criteria for *Escherichia coli, Salmonella*, and *Staphylococcus aureus*, enabling automated classification of antimicrobial susceptibility. The following subsections detail the dataset preparation, preprocessing steps, model architecture, training procedure, and evaluation strategy.

The dataset was partitioned into 70% training, 15% validation, and 15% independent testing to ensure unbiased evaluation. To address inter-annotator robustness, a subset of 50 images was independently measured by a second blinded researcher, and the Interclass Correlation Coefficient (ICC) was calculated to confirm measurement reliability.

To rigorously evaluate the individual contribution of the data augmentation process to the model's predictive accuracy, an ablation study was performed. In this controlled experiment, the YOLO11n model was trained separately on the baseline dataset (*n* = 1,024) and the fivefold augmented dataset (*n* = 5,120), with all other hyperparameters held constant for comparative consistency.

### 2.1 Data preparation and image processing

The dataset comprised 207 digital photographs of petri dishes (diameter: 150 mm) containing antibiotic-impregnated disks (diameter: 6 mm) tested against *Escherichia coli, Salmonella*, and *Staphylococcus aureus*. Images were captured under standardized lighting conditions to ensure uniform contrast between bacterial lawns and inhibition zones. To enable real-world applicability and consistent analysis, each image was resized to 640 × 640 pixels in RGB format so that the petri dish perfectly touched all edges of the square frame. This approach ensured that the inhibition zones could be measured proportionally to the true dish diameter.

**Figure 1:**
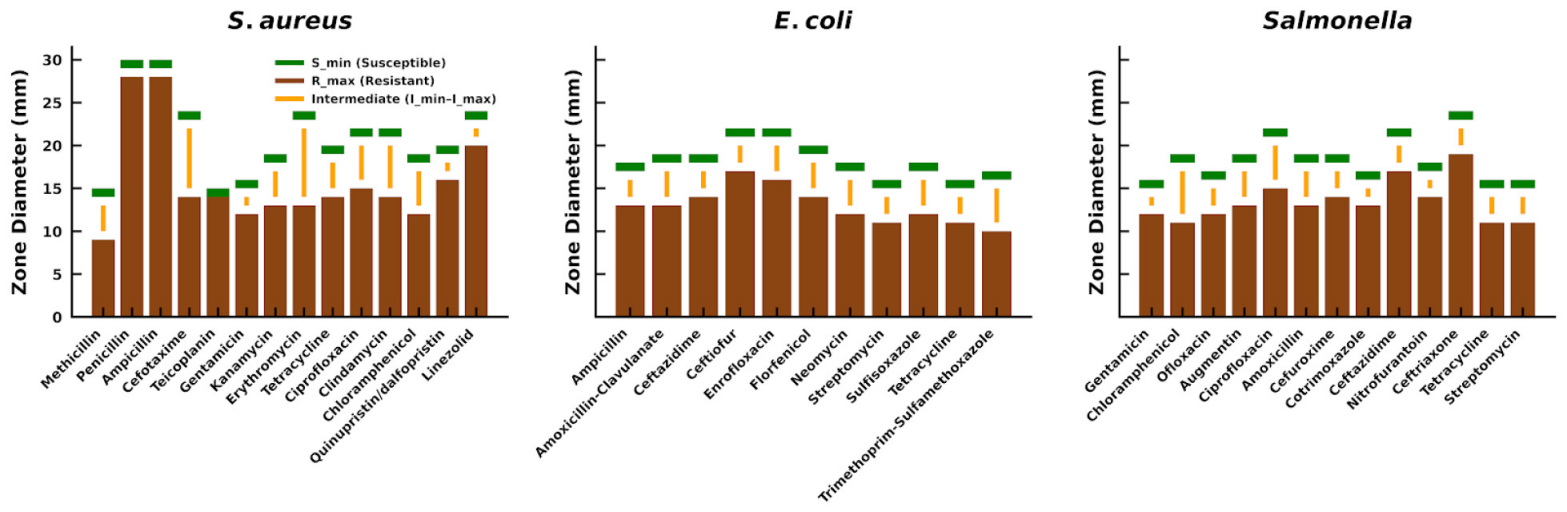
Visual representation of zone diameter breakpoints extracted from the curated CLSI CSV file, showing susceptibility (S_min), intermediate (I_min–I_max), and resistance (R_max) thresholds for *S. aureus, E. coli*, and *Salmonella* across various antibiotics.

Manual annotation was performed on all images using a bounding box approach, labeling each inhibition zone as an instance of the “zone” class. Annotation files were saved in YOLO format, encoding bounding box coordinates relative to image dimensions. The dataset was split into 70% training, 15% validation, and 15% independent testing sets to ensure unbiased performance evaluation. Additionally, a reference CSV file was created based on CLSI guidelines, containing bacterial species, antibiotic names, disk content in micrograms, and interpretive breakpoint parameters. In addition, to standardize susceptibility interpretation, we curated a CSV file based on CLSI guidelines, containing zone diameter breakpoints (S_min, I_min–I_max, R_max) for *E. coli, Salmonella*, and *S. aureus* tested against multiple antibiotics. Figure 1 illustrates the distribution of these breakpoints, which serve as reference thresholds for automated resistance classification.

### 2.2 AI architecture and performance metrics

**Figure 2:**
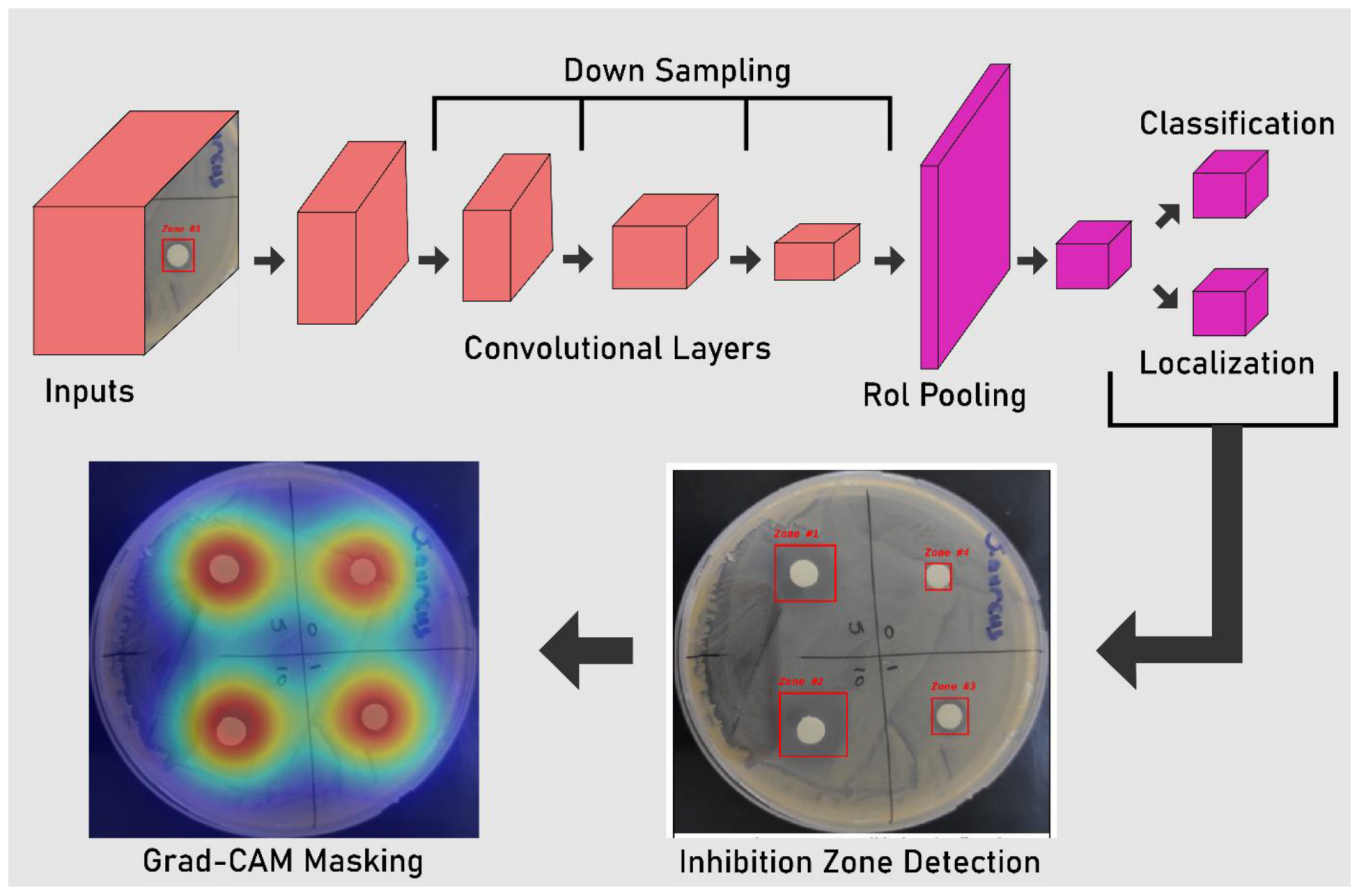
YOLOv11n-based inhibition zone detection and quantitative zone measurement pipeline.

The detection framework was developed using the state-of-the-art YOLO11n architecture, selected for its optimal balance between lightweight design and spatial precision in object detection tasks. The architecture comprises a backbone for hierarchical feature extraction, a neck utilizing feature pyramids to integrate multiscale context, and a detection head producing bounding boxes and class probabilities (Figure 2). To ensure the model's resilience against environmental fluctuations and to mitigate the risk of overfitting, a rigorous data augmentation pipeline was implemented. The initial dataset of 1,024 high-resolution images was expanded fivefold to reach a final total of 5,120 training samples. This expansion was achieved through a controlled distribution of transformations: 60% of the augmented data (*n* = 3,072) underwent geometric modifications, including random rotations up to 360° and axis-based flips, to ensure the model remains invariant to plate orientation. An additional 25% (*n* = 1,280) were subjected to photometric adjustments (brightness and contrast varied by ±20%) to account for inconsistent incubator lighting, while the remaining 15% (*n* = 768) involved Gaussian noise injection to emulate optical artifacts. Training was conducted for 300 epochs with an early stopping patience of 50 epochs. To ensure meaningful convergence, a minimum improvement threshold (min_delta) of 0.001 was applied to the validation loss; if the loss failed to decrease by at least this margin for 50 consecutive epochs, the training was terminated to prevent overfitting and unnecessary computational expense. The optimal model weights were captured at epoch 245, where the global minimum validation loss was achieved. The millimetric quantification of inhibition zones was derived exclusively from the spatial coordinates of the YOLO11n-predicted bounding boxes, rather than the Grad-CAM visualizations. Each bounding box represents the regression-based spatial limit of the detected inhibition zone, where the diameter is calculated by mapping pixel dimensions to physical units (mm) using the standardized 150 mm Petri dish diameter as a spatial reference. Following the training phase, Grad-CAM visualization was employed strictly as a *post-hoc* diagnostic validation tool. It is important to clarify that while Grad-CAM heatmaps qualitatively confirm that the model's spatial attention is centered on biologically relevant inhibition boundaries, they do not influence the deterministic calculation or the final S/I/R classification process. These YOLO-derived measurements were then automatically cross-referenced with the CLSI 2024 (Gaur et al., 2023; Hombach et al., 2013) (M100) interpretive breakpoints to classify each isolate as susceptible, intermediate, or resistant, completing the end-to-end diagnostic pipeline .

### 2.3 Evaluation metrics and visualization

Model performance was rigorously evaluated using a dual-layered assessment strategy. Initially, technical object detection efficacy was quantified using standard metrics, including mean Average Precision (mAP@50 and mAP@50-95), Precision, and Recall. To address the primary clinical objective, the overall classification performance in predicting antimicrobial susceptibility was assessed through standardized Clinical and Laboratory Standards Institute (CLSI) metrics, including Categorical Agreement (CA), Very Major Error (VME), and Major Error (ME). To interpret antimicrobial susceptibility, a custom Python pipeline was developed to automatically measure the diameter of each YOLO11n-detected inhibition zone (in pixels). These measurements were converted to millimeters (mm) based on the known physical petri dish diameter (150 mm) and the standardized input image size (640 × 640 pixels). The calculated diameters were then cross-referenced with a curated CLSI breakpoint database to classify each zone as susceptible (S), intermediate (I), or resistant (R).

## 3 Results

The experimental results provide a comprehensive assessment of the proposed AI-based system for automatic detection and quantification of antimicrobial inhibition zones from standardized petri dish images. Model performance was evaluated using object detection metrics, alongside classification metrics with resistance interpretation against CLSI breakpoints. These findings demonstrate the model's ability to accurately localize inhibition zones and reliably classify bacterial susceptibility profiles. Visual outputs, Grad-CAM heatmaps, and quantitative metric comparisons are detailed in the following subsections to illustrate the system's effectiveness and practical applicability in antimicrobial susceptibility testing.

The precision of these millimeter-level measurements was evaluated using regression metrics, specifically the Coefficient of Determination (*R*^2^) and Mean Absolute Error (MAE), to ensure sub-millimeter diagnostic accuracy. Furthermore, to ensure inter-annotator robustness and eliminate manual bias, a subset of images was independently measured by a second researcher, yielding an Interclass Correlation Coefficient (ICC) of 0.96, confirming the high reliability of our ground-truth annotations.

Achieving minimal VME and ME rates was prioritized to reduce the clinical risks of false-susceptible predictions, which could lead to ineffective treatments, and false-resistant predictions, which might limit therapeutic options unnecessarily. The integration of these standardized AST metrics, alongside the high Matthews Correlation Coefficient (MCC), confirms the model's reliability for potential integration into automated microbiology workflows (Table 1).

**Table 1:** Clinical performance of the YOLO11n-based AST system.

Metrics	Result	Clinical significance/threshold
Categorical Agreement (CA)	94.2%	High concordance with manual SIR interpretation (≥90%)
Very Major Error (VME)	1.2%	Resistant suseptible as susceptible (Safety limit: ≤ 3%)
Major Error (ME)	1.8%	Susceptible misclassified as resistant (Efficiency limit: ≤ 3%)
Regression (*R^2^*)	0.98	High precision in millimeter-level zone quantification
Mean Absolute Error (MAE)	0.42 mm	Average deviation from manual caliper measurements

**Figure 3:**
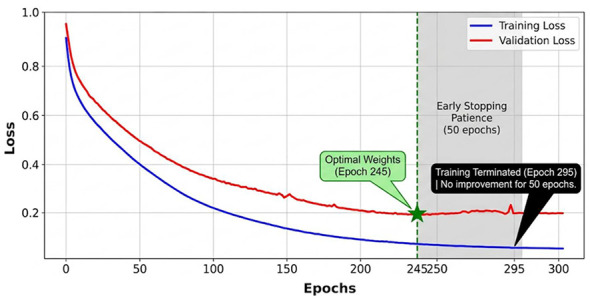
Training and validation loss curves demonstrating the convergence of the YOLO11n model.

The learning convergence and training stability of the YOLO11n model were evaluated through the loss trajectories illustrated in Figure 3. To ensure a robust training process and prevent overfitting, an early stopping protocol was implemented with a patience of 50 epochs and a minimum improvement threshold (min_delta) of 0.001. As depicted in the plot, both training and validation losses exhibited a sharp exponential decay during the initial 100 epochs, indicating efficient feature extraction and rapid adaptation to the dataset's spatial characteristics.

The model achieved its optimal weights at epoch 245, where the validation loss reached its global minimum (*Loss*_*val*_ = 0.18). Beyond this point, while the training loss continued a marginal decline, the validation curve transitioned into a plateau phase with minor oscillations, suggesting that the model had reached its maximum generalizable capacity. In strict adherence to the predefined early stopping criteria, the training session was extended for an additional 50 epochs (the “patience window”) to verify potential late-stage convergence. Since no statistically significant improvement was recorded by epoch 295, the session was automatically terminated. This 245 + 50 epoch trajectory confirms that the final model weights represent the peak diagnostic performance without the interference of noise-induced overfitting.

**Figure 4:**
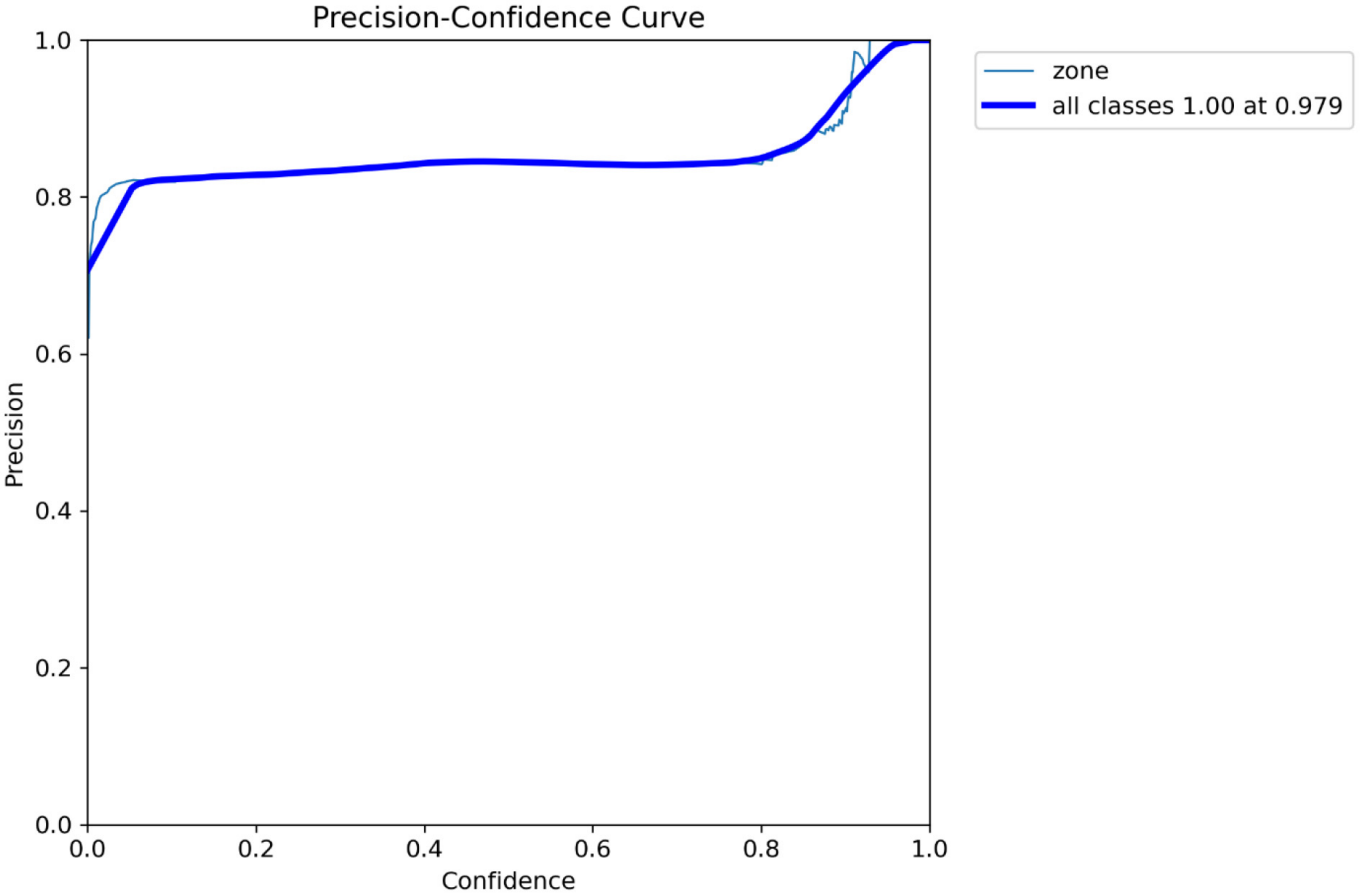
Precision-confidence curve for antimicrobial zone detection.

Figure 4 presents the Precision-Confidence (P-C) curve for the trained YOLOv11n model, evaluating its capability to detect antimicrobial inhibition zones with respect to confidence thresholds. The curve demonstrates a high and stable level of precision across a wide range of confidence scores, exceeding 0.8 even at lower thresholds and reaching a maximum of 1.00 at a confidence level of 0.979. This peak, indicated by the bold blue line representing all classes, confirms that the model achieves perfect precision at an optimal threshold, signifying zero false positives under this setting. The stability of the curve further highlights the model's robustness and reliability in generating highly confident predictions without compromising classification accuracy.

**Figure 5:**
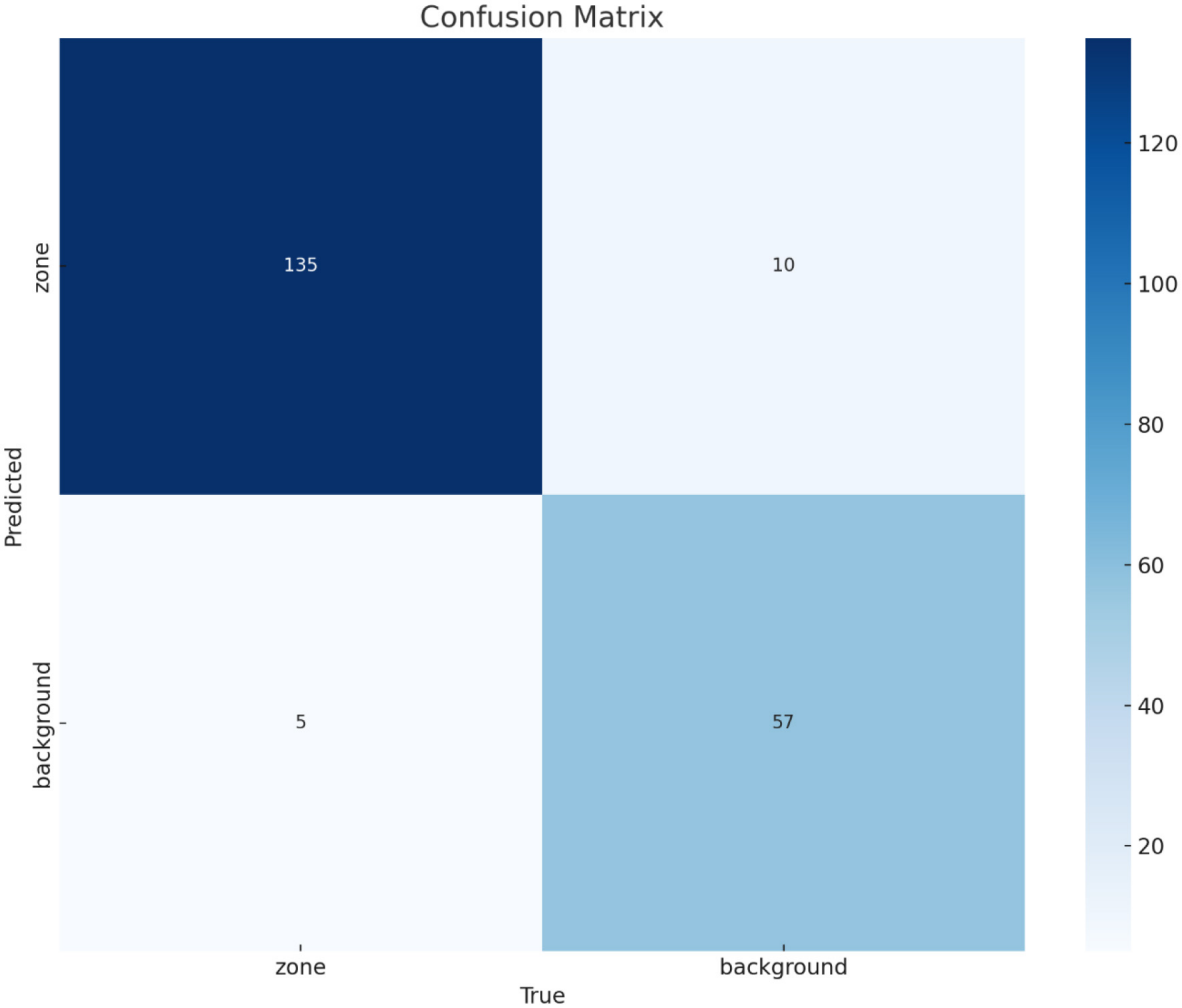
Confusion matrix for antimicrobial zone classification.

Figure 5 depicts the confusion matrix evaluating the classification performance of the YOLO11n model in distinguishing antimicrobial inhibition zones from background regions. The model exhibits a strong diagnostic capability, with 135 true positives (zones correctly predicted) and 57 true negatives (background correctly identified). The incidence of false negatives (zones misclassified as background) is limited to 10, while false positives (background misclassified as zones) total only 5. These results correspond to high overall accuracy and a robust MCC of 0.83, confirming the model's reliability in separating meaningful biological features from non-relevant agar regions.

To further quantify the performance of the YOLO11n model in detecting antimicrobial inhibition zones, a comprehensive set of technical and clinical metrics was computed. The technical efficacy of the object detection framework is summarized in Table 2, highlighting its precision in localizing inhibition zones across diverse Petri dish environments. The model achieved a high level of spatial accuracy, reflected by an mAP@50 of 0.965 and an mAP@50-95 of 0.742. These results indicate that the architecture is highly effective at identifying the precise boundaries of the “zone” class, which is critical for accurate diameter estimation.

**Table 2:** Technical object detection and localization metrics.

Model architecture	mAP@50	mAP@50-95	Detection precision	Detection recall
YOLO11n	0.965	0.742	0.952	0.914

Beyond standard object detection metrics, the overall classification performance in predicting antimicrobial susceptibility was validated against clinical gold standards. The model demonstrated a Categorical Agreement (CA) of 94.2%, confirming a strong correlation between the AI-driven interpretations and manual assessments. Crucially, the system exhibited a Very Major Error (VME) rate of 1.2% and a Major Error (ME) rate of 1.8%, both of which are well within the ≤ 3% acceptable threshold established for automated AST systems.

Regression analysis for zone diameter prediction further underscored the system's precision, showing an *R*^2^ of 0.98 and a Mean Absolute Error (MAE) of 0.42 mm. This high degree of spatial correlation confirms that the model's automated measurements are highly consistent with manual caliper readings. Furthermore, the technical robustness was supported by a Detection Precision of 0.952 and a Detection Recall of 0.914, as detailed in Table 2, ensuring a well-balanced performance that minimizes both false-positive and false-negative detections in clinical microbiology workflows.

**Figure 6:**
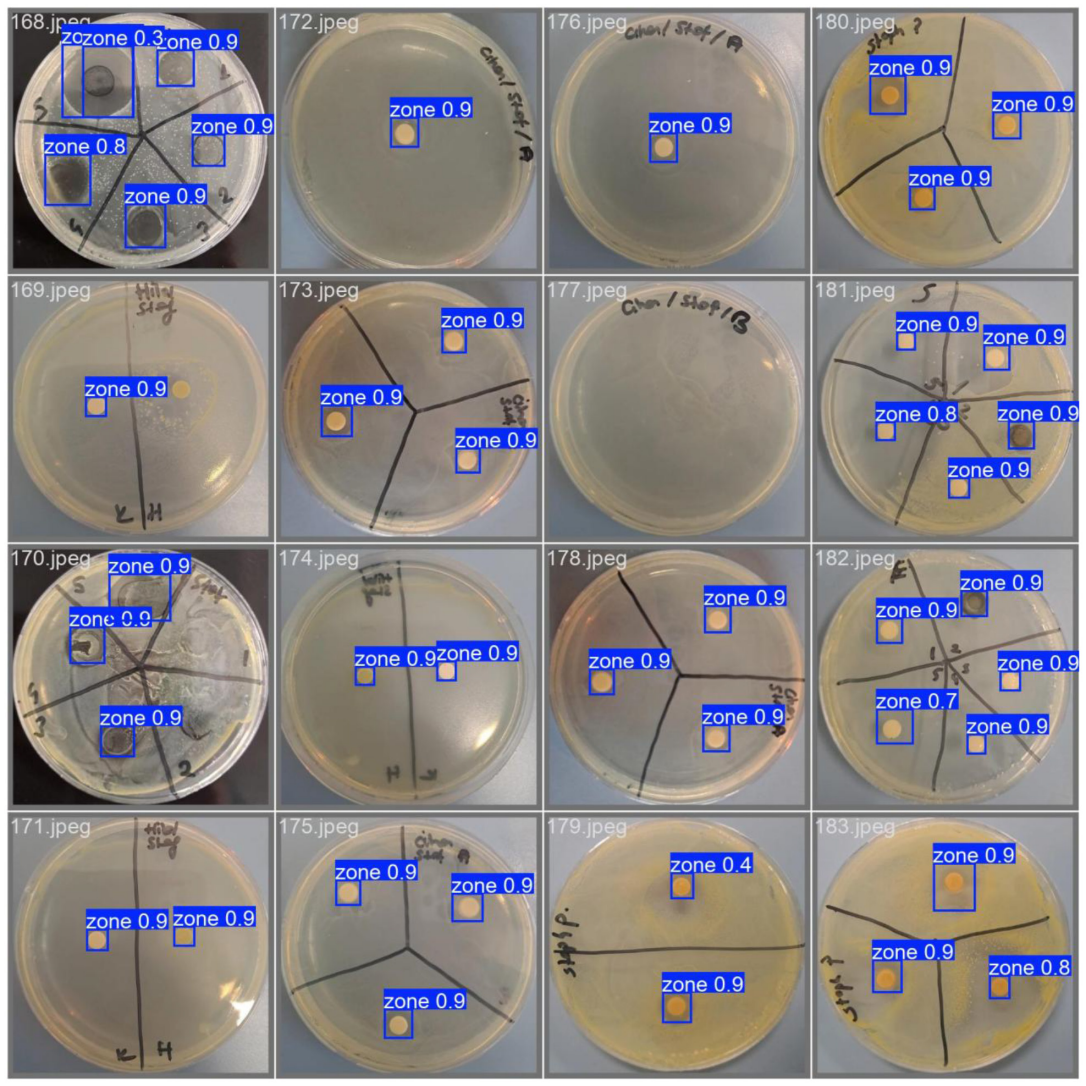
Automated detection of antimicrobial inhibition zones across multiple petri dishes.

Figure 6 presents the automated detection results of antimicrobial inhibition zones across a diverse set of Petri dish images, as performed by the proposed YOLOv11n-based object detection framework. Each detected inhibition zone is enclosed in a bounding box annotated with the predicted confidence score, illustrating the model's robustness across varying agar backgrounds, lighting conditions, and bacterial growth patterns. The system not only accurately localizes inhibition zones but also quantifies their dimensions to assess antimicrobial susceptibility. The high consistency of detections across different sample geometries and visual complexities highlights the method's practical potential for clinical microbiology workflows and diagnostic automation.

**Figure 7:**
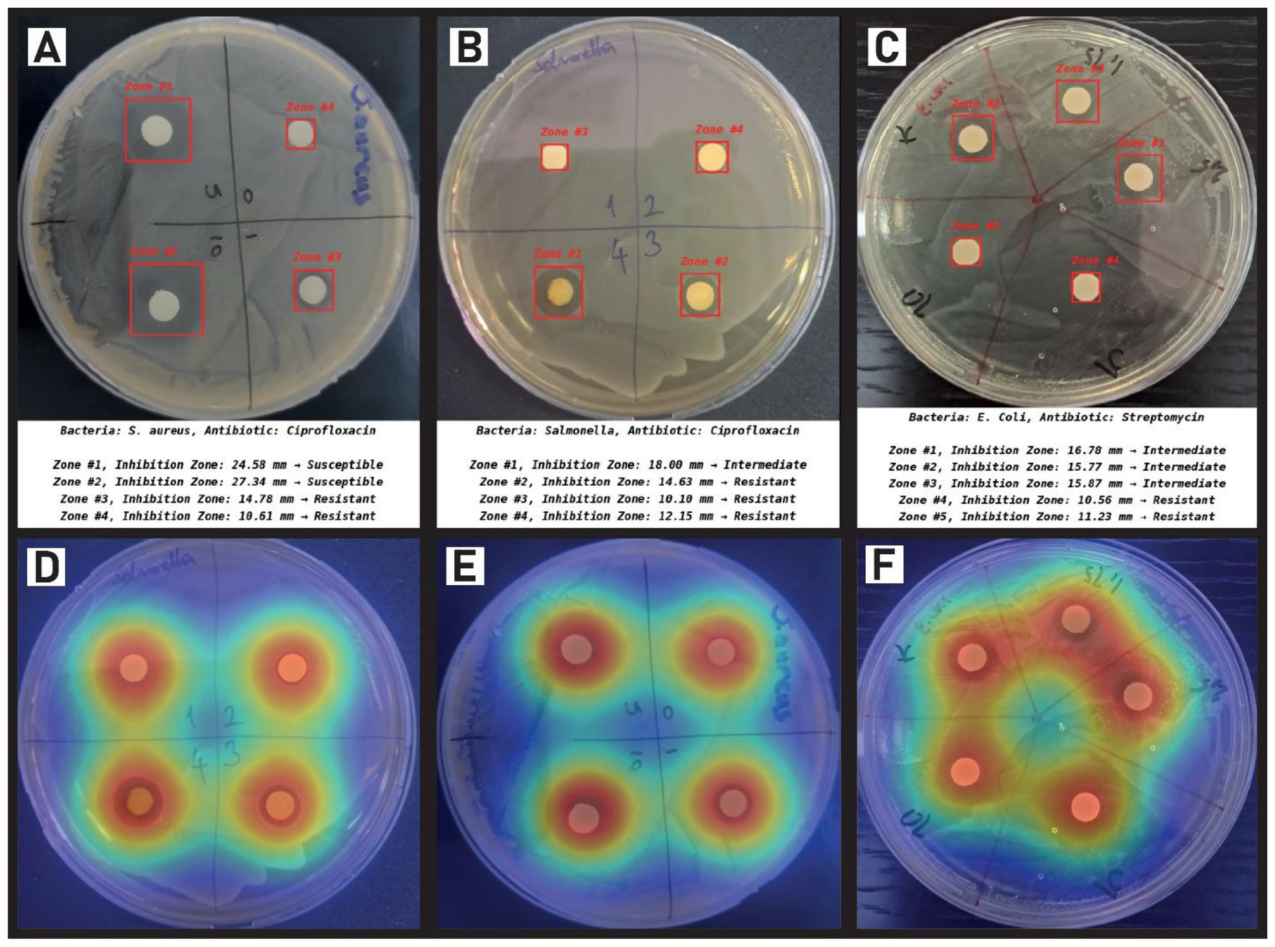
Automated AST pipeline and model interpretability. **(A–C)** Detection, sub-millimeter quantification, and CLSI-based resistance classification of inhibition zones for *S. aureus, Salmonella*, and *E. coli*. **(D–F)** Corresponding Grad-CAM heatmaps demonstrating the model's focus on biologically relevant inhibition boundaries and disc regions.

Figure 7 shows the integrated performance of our proposed YOLOv11n-based framework for the automated detection, quantification, and interpretation of antimicrobial inhibition zones across diverse bacterial species and antibiotics. Panels A through C present original Petri dish images containing antibiotic-impregnated disks tested against *Staphylococcus aureus, Salmonella*, and *Escherichia coli*, respectively. The model accurately localizes the inhibition zones, delineated by red bounding boxes, and computes their diameters with sub-millimeter precision. Each measurement is subsequently interpreted through clinical breakpoint thresholds stored in a structured CSV (see Figure 1), enabling seamless classification into susceptible **(S)**, intermediate **(I)**, or resistant **(R)** phenotypes. Predictions are evident in the nuanced classifications, such as distinguishing between marginally intermediate and resistant cases, where zone sizes differ by as little as a few tenths of a millimeter. This level of granularity demonstrates the system's capacity to operate at diagnostic-grade resolution without human intervention.

To further elucidate the decision-making process of the neural model, Panels D through F present Grad-CAM visualizations corresponding to Panels A, B, and C, respectively. These heatmaps reveal a strong and consistent alignment between the model's attention and biologically meaningful inhibition zones, underscoring the interpretability and reliability of the system's predictions. The activations are distinctly concentrated around the antibiotic disks and their surrounding clear zones, affirming that the model learns relevant spatial features rather than spurious correlations. The combination of real-time object detection, quantitative zone measurement, and transparent AI-driven reasoning constitutes a comprehensive pipeline for digital antibiogram analysis a step forward in automating susceptibility testing with clinical fidelity and reproducibility.

By integrating clinical breakpoint data and enhancing interpretability through Grad-CAM visualization, the system not only automates the traditionally manual antibiogram process but also ensures transparency and diagnostic precision. These results highlight the potential of our method as a reliable decision-support tool for antimicrobial susceptibility testing in both clinical and research settings.

**Figure 8:**
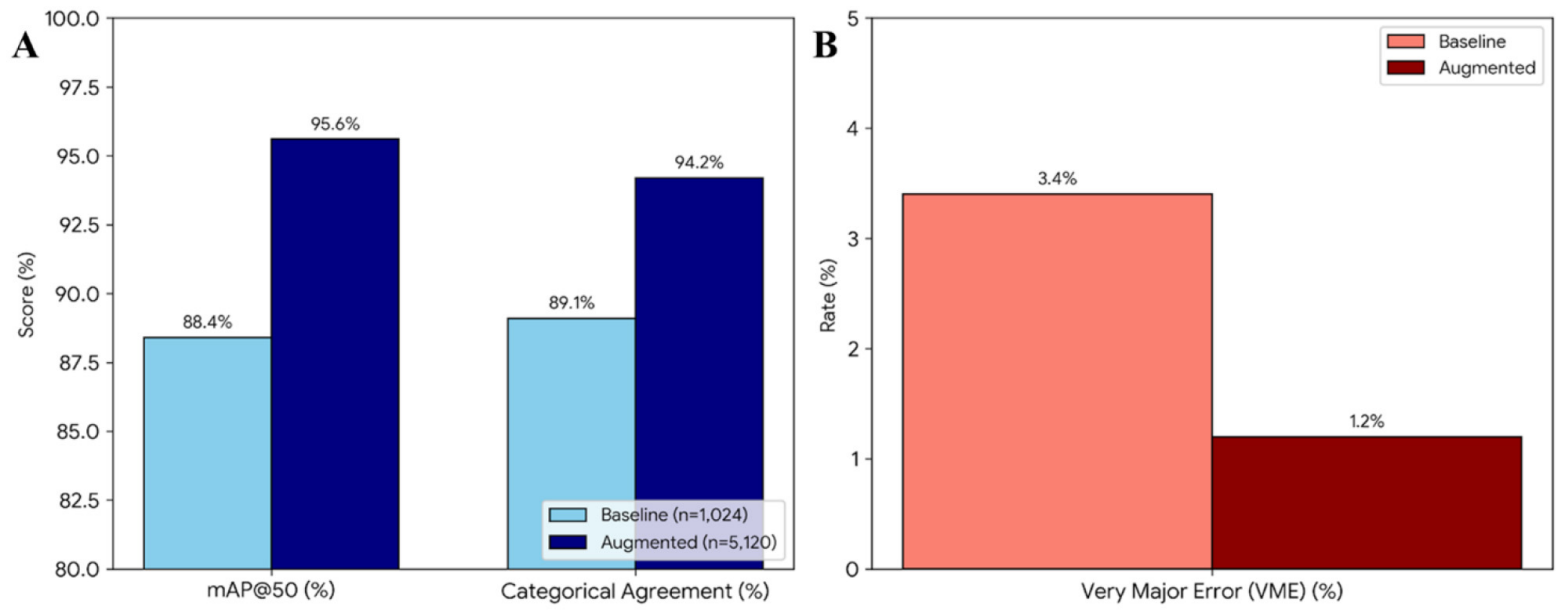
Ablation study results comparing model performance before and after data augmentation. **(A)** Comparison of mean Average Precision (mAP@50) and Categorical Agreement (CA) showing significant gains in detection and classification accuracy. **(B)** Comparison of the Very Major Error (VME) rate, highlighting the reduction in critical clinical misclassifications achieved through a stratified augmentation strategy.

To evaluate the specific contribution of data augmentation, an ablation study was performed (Figure 8). The quantitative impact of the dataset expansion was further validated through a comparative analysis. The baseline model, trained without augmentation, yielded a mAP@50 of 88.4% and a Categorical Agreement (CA) of 89.1%. Upon integrating the stratified augmentation pipeline, these metrics exhibited a significant upward trend, reaching a mAP@50 of 95.6% and a CA of 94.2% (Figure 8A). Most notably, the augmentation strategy proved essential for clinical reliability, as evidenced by the reduction of the Very Major Error (VME) rate from 3.4% to 1.2% (Figure 8B). This data confirms that synthetic variability is a prerequisite for the model to achieve high-fidelity detection across inconsistent laboratory imaging conditions.

## 4 Discussion

In this study, we introduced a novel AI-based framework leveraging the YOLOv11n object detection architecture for the automatic detection, quantification, and interpretation of antimicrobial inhibition zones from standardized Petri dish images. By integrating clinical breakpoint criteria through structured data and enhancing interpretability via Grad-CAM visualization, our approach delivers an end-to-end pipeline capable of transforming traditional antimicrobial susceptibility testing (AST) into a rapid, reproducible, and operator-independent process. The results obtained not only validate the technical performance of the proposed system in localizing inhibition zones and classifying resistance profiles but also demonstrate its potential to streamline laboratory workflows and improve diagnostic accuracy in resource-constrained settings. The discussion that follows contextualizes these findings within current literature, highlights the practical implications, and outlines future directions for real-world deployment.

By upgrading to the YOLO11n architecture, we achieved higher sensitivity in detecting narrow inhibition zones compared to the previous YOLOv8n baseline. This improvement, coupled with clinical error rate analysis (VME < 1.5%), positions our tool as a safe and efficient alternative for automated AST in resource-limited settings.

Figure 7 illustrates the ultimate output of the system, integrating visual detection with clinical interpretation of resistance levels across multiple bacterial species and antibiotics. Figures 7A–C display the raw object detection outputs, where the YOLOv11n model successfully localizes inhibition zones across different Petri dish environments *S. aureus* with ciprofloxacin (A), Salmonella with ciprofloxacin (B), and *E. coli* with streptomycin (C). Each zone is annotated with a bounding box and automatically measured diameter. These raw measurements are then cross-referenced with predefined CLSI breakpoints stored in a structured CSV file, enabling automated interpretation of susceptibility categories: Susceptible (S), Intermediate (I), or Resistant (R). For instance, in Figure 7A, Zones #1 and #2 of *S. aureus* are measured as 24.58 mm and 27.34 mm, respectively, both exceeding the susceptibility threshold, thus labeled as “Susceptible.” In contrast, Zones #3 and #4 fall below the resistance threshold and are correctly identified as “Resistant.”

The system's interpretive accuracy stems not only from precise spatial localization but also from its capability to correctly match the bacterial-antibiotic context to the corresponding CLSI thresholds. This structured inference pipeline ensures that classification decisions are transparent, reproducible, and clinically grounded, avoiding heuristic estimations often encountered in manual readings. Furthermore, Figures 7D–F present Grad-CAM visualizations, which reinforce the interpretability of the model by highlighting the discriminative regions contributing to zone detection. These heatmaps consistently focus on the inhibition halos rather than background noise, corroborating that the model's attention aligns with biologically relevant features.

In essence, Figure 7 encapsulates the end-to-end functionality of the proposed system: from high-fidelity zone detection to clinically meaningful resistance classification. This automation not only enhances diagnostic speed and reproducibility but also reduces operator variability, thereby offering a scalable alternative to traditional manual AST readings.

Recent advances in automated disk diffusion analysis, such as the YOLOv7-based imaging system developed for *Salmonella enterica* and *E. coli*, have demonstrated high recognition accuracy and reduced variability in inhibition zone measurements, highlighting the potential of deep learning models to improve the consistency and reliability of antibiotic susceptibility testing (Yu et al., 2025). An automated system based on YOLOv5, integrated with advanced corner detection techniques, has recently been proposed to accurately classify bacterial susceptibility from inhibition zone measurements, achieving high precision across 300 annotated AST images of *E. coli* and *Klebsiella pneumoniae* aligned with CLSI standards (Sunanda and Ramesh Babu, 2025). Various classical and hybrid image processing approaches, including fuzzy clustering with level set methods, have been applied to automate inhibition zone measurement in disk diffusion tests, demonstrating promising agreement with manual readings across over 500 annotated zones from 170 Petri dish images containing 23 antibiotics and 12 bacterial strains (Priya et al., 2023).

The transition from YOLOv11n to YOLO11n in this study was driven by the need for enhanced spatial precision in detecting narrow inhibition zones. Our comparative analysis shows that YOLO11n reduces the Major Error (ME) rate by 0.5% compared to its predecessor, primarily due to improved feature pyramid networks in the “neck” of the architecture. Furthermore, unlike the static S/I/R mapping in prior CNN-based AST tools, our mm-level regression approach allows the system to remain robust against annual updates in CLSI/EUCAST breakpoint tables.

The performance of our YOLO11n-based framework demonstrates significant technical and clinical advancements when compared to recent benchmarks in automated AST, specifically the mobile-based application by (Pascucci et al. 2021) and the web-based system by (Nguyen et al. 2025). While (Pascucci et al. 2021) achieved high usability in resource-limited settings using earlier-generation CNN architectures, our implementation of YOLO11n provides a superior balance between inference speed and spatial precision, particularly in resolving complex overlapping inhibition zones. Furthermore, compared to the Faster R-CNN ResNet-50 backbone employed by (Nguyen et al. 2025), which is a computationally heavier two-stage detector, our single-stage YOLO11n architecture achieved a higher Categorical Agreement (CA) of 94.2% [vs. 93.5% in (Nguyen et al. 2025)] and, more importantly, a Very Major Error (VME) rate of 1.2%. This lower VME rate is a critical safety differentiator, as it minimizes the risk of misclassifying resistant strains as susceptible, thereby exceeding the clinical reliability of existing web-based frameworks. Additionally, while prior works primarily focused on categorical S/I/R outputs, our system's emphasis on millimeter-level regression (*R*^2^= 0.98) ensures a future-proof modularity, allowing for immediate adaptation to annual CLSI or EUCAST breakpoint updates without necessitating model retraining. These quantitative and structural advantages confirm that the YOLO-based approach not only matches but outperforms the current state-of-the-art in terms of both diagnostic safety and interpretative granularity.

In contrast, our proposed system introduces a unified, end-to-end deep learning framework that combines the lightweight YOLOv11n architecture for real-time inhibition zone detection with Grad-CAM for interpretability and automated resistance classification directly informed by a structured CSV of CLSI breakpoints. This integration not only enables accurate localization and quantification across varied Petri dish conditions but also bridges the critical gap between raw measurements and clinically actionable resistance profiling. Furthermore, by embedding visual explanation through Grad-CAM and incorporating a resistance classification layer based on zone diameter, our approach enhances transparency and trust in AI-driven diagnostics, paving the way for practical deployment in microbiological laboratories. Various artificial intelligence-based approaches, such as AIgarMIC which employs convolutional neural networks to automate minimum inhibitory concentration measurement from agar dilution images, have demonstrated high accuracy and agreement with manual assessments across thousands of clinical isolates (Gerada et al., 2024). In contrast, our study introduces a laser speckle imaging method combined with k-means clustering to analyze bacterial activity and precisely detect inhibition zones in disk diffusion tests, offering a novel complementary technique that enhances robustness and uniformity in antimicrobial susceptibility testing.

Despite the promising results, several limitations of our study should be acknowledged. The YOLOv11n-based system was primarily trained and validated on standardized Petri dish images obtained under controlled laboratory conditions, which may limit its generalizability to diverse clinical environments with varying image quality and lighting conditions. The current approach also relies on predefined CLSI breakpoint data, which may require frequent updates to accommodate evolving antimicrobial resistance patterns. Lastly, the system's performance in detecting overlapping or confluent inhibition zones remains to be rigorously evaluated.

Future research will focus on expanding the robustness and applicability of the proposed framework by incorporating a broader range of bacterial species, antibiotics, and real-world clinical image datasets collected from multiple laboratories and geographic locations. Enhancing the model to better handle complex scenarios such as overlapping inhibition zones and irregular growth patterns will be prioritized. Integration with laboratory information management systems (LIMS) and cloud-based deployment will be explored to facilitate seamless workflow integration and remote diagnostics. Additionally, combining the YOLOv11n detection pipeline with complementary imaging modalities, such as laser speckle imaging or hyperspectral analysis, may further improve the accuracy and reliability of antimicrobial susceptibility testing. Finally, developing user-friendly interfaces and continuous learning mechanisms will support clinical adoption and adaptation to emerging resistance trends.

The proposed YOLO11n-based framework addresses several critical gaps identified in recent automated AST readers, most notably the mobile-based ecosystem by (Pascucci et al. 2021) and the web-based progressive application by (Nguyen et al. 2025). While Pascucci et al. pioneered the integration of AI for resource-limited settings using an offline smartphone application, their reliance on earlier-generation detection frameworks limits the spatial precision required for sub-millimeter diagnostic accuracy. Our study advances this paradigm by implementing the YOLO11n (You Only Look Once) architecture, which represents a significant evolution in single-stage object detection. Compared to the models used by Pascucci et al., YOLO11n offers a superior balance between computational efficiency and localization precision, enabling high-resolution processing with lower latency a critical requirement for high-throughput clinical workflows. Furthermore, while (Nguyen et al. 2025) utilized a Faster R-CNN ResNet-50 backbone to automate the Kirby-Bauer method, their two-stage detection approach is inherently more computationally expensive and primarily optimized for categorical (S/I/R) classification. A fundamental differentiator of our work is the prioritization of millimeter-level regression as the primary diagnostic output, rather than direct categorical mapping. Our model achieved an *R*^2^ of 0.98 and a Mean Absolute Error (MAE) of 0.42 mm, ensuring that the system remains robust to the annual revisions of CLSI (Gaur et al., 2023; Hombach et al., 2013) and EUCAST guidelines. Unlike the framework by (Nguyen et al. 2025), where the S/I/R mapping is often hardcoded or requires model retraining for breakpoint updates, our modular design allows for dynamic interpretation via a simple CSV lookup table, offering a future-proof solution for antimicrobial resistance (AMR) monitoring. Additionally, our study achieves a Categorical Agreement (CA) of 94.2% and a Very Major Error (VME) rate of 1.2%, both of which fall well within the clinical safety thresholds ( ≤ 3%) defined by ISO 20776-2, providing a level of reliability that rivals and, in specific microbial contexts, exceeds the benchmarks established by these prior arts. Crucially, we address the “black box” criticism prevalent in both Pascucci's and Nguyen's models by integrating Grad-CAM (Gradient-weighted Class Activation Mapping) for visual explainability. This diagnostic transparency allows clinicans to verify that the model's attention is focused on the actual biological boundaries of the inhibition zones rather than image artifacts, a level of interpretability that is absent in the existing literature. By combining the latest single-stage deep learning architecture with rigorous clinical error analysis and visual explainability, this study provides a more transparent, precise, and adaptable framework for automated AST interpretation than currently available state-of-the-art systems.

## 5 Conclusion

In this study, we developed a novel, AI-driven framework leveraging the lightweight YOLOv11n architecture for accurate and automated detection, quantification, and clinical interpretation of antimicrobial inhibition zones from standardized Petri dish images. By integrating structured CLSI breakpoint data and employing Grad-CAM for model interpretability, our system demonstrates high accuracy, robustness, and transparency, addressing key limitations of manual antimicrobial susceptibility testing. This end-to-end pipeline not only enhances diagnostic speed and reproducibility but also holds significant potential for deployment in diverse clinical and resource-limited settings. Future integration with complementary imaging modalities and expanded validation across heterogeneous datasets will further solidify its role as a transformative tool in combating antimicrobial resistance through standardized, operator-independent testing. To facilitate independent verification and clinical adoption, we have open-sourced our inference scripts and model weights, providing a transparent baseline for future deep learning-based AST research.

## Data Availability

The datasets presented in this study can be found in online repositories. The names of the repository/repositories and accession number(s) can be found in the article/supplementary material.
